# miR-15b/16 protects primary human retinal microvascular endothelial cells against hyperglycemia-induced increases in tumor necrosis factor alpha and suppressor of cytokine signaling 3

**DOI:** 10.1186/s12974-015-0265-0

**Published:** 2015-03-04

**Authors:** Eun-Ah Ye, Jena J Steinle

**Affiliations:** Department of Anatomy and Cell Biology, Wayne State University, 9314 Scott Hall, 48201 Detroit, MI USA; Department of Ophthalmology, Wayne State University, 9314 Scott Hall, 48201 Detroit, MI USA

**Keywords:** miR-15b/16, REC, TNFα, SOCS3, Insulin signaling, IGFBP-3

## Abstract

**Background:**

Mechanisms underlying the pathology of diabetic retinopathy are still not completely understood. Increased understanding of potential cellular pathways responsive to hyperglycemia is essential to develop novel therapeutic strategies for diabetic retinopathy. Emerging evidence shows the impact of microRNA (miR) as a potential novel therapeutic target. The purpose of our study was to test the hypothesis that miR-15b and miR-16 are altered by hyperglycemia in retinal endothelial cells (REC), and that miR-15b/16 play key roles in regulating insulin signaling through a reduction in TNFα- and suppressor of cytokine signaling 3 (SOCS3)-mediated insulin resistance pathways.

**Methods:**

Human REC were maintained in normal (5 mM) glucose or transferred to high-glucose medium (25 mM) for 3 days. REC were transfected with miRNA mimics (hsa-miR-15b-5p and hsa-miR-16-5p) 48 h before cell harvest. A final concentration of 30 nM was used when transfected separately (miR-15b and miR-16) and 15 nM was used in combination (miR-15b + miR-16). A negative control group was treated with an equal concentration of a mimic negative control. The levels of miRNA overexpression were verified using quantitative reverse transcription-polymerase chain reaction and real-time PCR. Western blot analyses were performed to study the levels of phosphorylated Akt (Serine 473), Akt, SOCS3, insulin receptor, phosphorylated insulin receptor (tyrosine 1150/1151), and insulin receptor phosphorylated on Tyr960. In addition, ELISA was used to examine cleaved caspase 3 and TNFα. Analyses were done using unpaired Student *t* test. Data are presented as mean ± S.E.M.

**Results:**

We demonstrated that the expression of miR-15b and miR-16 was reduced in human REC cultured in hyperglycemia. Overexpression of miR-15b and/or miR-16 reduced TNFα and SOCS3 levels, while increasing insulin-like growth factor binding protein-3 (IGFBP-3) levels and the phosphorylation of insulin receptor (IR)^Tyr1150/1151^ in REC cultured in hyperglycemia. These, in turn, led to an increase of Akt phosphorylation and decreased cleavage of caspase 3.

**Conclusions:**

miR-15b and miR-16 play a role in the inhibition of insulin resistance via reduced TNFα and SOCS3 signaling and increased IGFBP-3 levels, resulting in REC protection from hyperglycemia-induced apoptosis. This outcome suggests that both miR-15b and miR-16 are potential therapeutic targets for therapeutics for the diabetic retina.

## Background

Diabetic retinopathy is the leading cause of blindness in adults in the US [1]. Retinopathy results in vision loss for >10,000 people with diabetes every year and is the most common diabetic complication of the microvasculature [2]. It has been reported that almost all patients with type 1 diabetes mellitus (T1DM) and more than 60% of people with T2DM have retinopathy after 20 years of diabetes [3]. Although much work has been completed on potential causes for diabetes-induced retinal damage, the mechanisms of diabetic retinopathy are still not completely understood.

It has been known that hyperglycemia is a key risk factor that contributes to the onset and progression of diabetic retinopathy [4-6]. Chronic hyperglycemia can damage a variety of cell types and result in dysfunction and failure of many organs, including the eyes [7]. Increased understanding of the pathological pathways associated with high glucose is critical, as few therapeutics exist for diabetic retinopathy until late in the disease progression where laser photocoagulation can be used in some patients. Increased understanding of potential cellular pathways responsive to hyperglycemia is essential to develop novel therapeutic strategies for diabetic retinopathy.

One potential mechanism is changes in key microRNAs (miR) in response to exposure to hyperglycemia. As an emerging key regulator of gene expression, miRNAs have been identified and investigated for approximately 10 years. The great potency of miRNA (small noncoding RNA molecules of 21 to 23 nucleotides) in regulating a variety of cellular activities, including cellular development, proliferation, differentiation, and death, has been demonstrated [8] as well as the use of miRNA as a potential biomarker for diagnosing diseases [9-11]. miRNA expression and their functions are tissue- and cell-type-specific. Diabetes mellitus is associated with the altered expression of several miRNAs in insulin-target tissues, as well as in insulin-secreting cells [12]. A group of miRNAs, including miR-15b, miR-16, miR-21, miR-93, miR-132, miR-146, and miR-200, have been identified as having altered expression in diabetic retinopathy [13-15]. To date, however, there is only a small number of studies completed to establish the downstream cellular signaling of miRNA to understand how specific miRNA may affect the pathological mechanisms common in diabetic retinopathy. Recently, the potential of miR-15b and miR-16 have been investigated in diabetic retinopathy. The expression of miR-15b and miR-16 was identified in human umbilical vein endothelial cells (HUVEC), with overexpression of miR-15b or miR-16 playing a role in the inhibition of angiogenesis [16,17]. Another work has reported increased miR-15b and miR-16 expression in rat retinal endothelial cells (REC) of streptozotocin (STZ)-induced diabetic rats [14]. However, little is known about the expression of miR-15b and miR-16 in human REC and its potential role in the downstream cellular signaling associated with the pathogenesis of diabetic retinopathy.

In order to best understand a potential role for key miRNAs in diabetic retinopathy, it is first important to establish whether specific miRNAs change in cell types critical to retinopathy. REC, as one of the crucial cell types substantially affected in diabetic retinopathy, have been intensively studied in our lab, as well as others, and the role and molecular mechanisms underlying diabetic retinopathy have been deeply investigated [18-25]. We previously reported that TNFα levels are increased in REC under high-glucose conditions. TNFα increased suppressor of cytokine signaling 3 (SOCS3) signaling, as well as insulin receptor substrate 1 (IRS-1)^Ser307^ and insulin receptor (IR)^Tyr960^ phosphorylation, leading to inhibition of normal insulin signaling under high-glucose conditions [22]. miR-15b and miR-16 are predicted to target a number of molecules, including downstream protein involved in insulin signaling (targetscan.org). In the present study, we tested the hypothesis that miR-15b and miR-16 levels are altered by exposure to hyperglycemia in REC, and that miR-15b/16 play key roles in regulating insulin signaling through a reduction in TNFα- and SOCS3-mediated insulin resistance pathways.

## Materials and methods

### Cell culture

Human REC were acquired from Cell Systems Corporation (CSC, Kirkland, WA). Cells were grown in M131 medium containing microvascular growth supplement (Invitrogen), 10 μg/ml gentamycin, and 0.25 μg/ml amphotericin B. For experiments, the cells were maintained in normal (5 mM) glucose or transferred to a high-glucose medium (25 mM) (Cell Systems) for 3 days. Only primary cells within passage 5 were used. Cells were quiesced by incubating in high- or normal-glucose medium without growth supplementation for 20 h and used to perform the experiments.

### Cell transfection with microRNA mimics

REC were transfected with miRNA mimics (hsa-miR-15b-5p and hsa-miR-16-5p) (Invitrogen, Carlsbad, CA) using Oligofectamine (Invitrogen) following manufacturer instructions. miR transfection was performed 48 h before cell harvest. A final concentration of 30 nM was used when transfected separately (miR-15b and miR-16) and 15 nM was used in combination (miR-15b + miR-16). The negative control group was treated with an equal concentration (30 nM) of a Mimic Negative Control (Invitrogen). Other control groups, normal glucose (NG) and high glucose (HG), were treated with 0 nM mimic with Oligofectamine. The levels of miRNA overexpression were verified using quantitative reverse transcription-polymerase chain reaction and real-time PCR.

### Real-time quantitative PCR

The total RNA was isolated and purified using the TRIzol method, and the purity and quantity of RNA were measured using Nanodrop (ND-1000). For polyA tailing reverse-transcriptase PCR, 5 μg of total RNA was treated with DNase I for 15 min at room temperature (Promega) and then polyA using (polyA) polymerase (NEB; Ipswich, MA) was added at 37°C for 1 h. The final reaction mixtures were extracted with phenol/chloroform, precipitated with isopropanol, and re-dissolved in 25 μl diethylpyrocarbonate (DEPC)-treated water. PolyA-tailed RNA (6 μl) was reverse-transcribed into first-strand cDNA using Superscript II reverse transcriptase (Invitrogen) with the oligo-dT adapter primer 5′GCGAGCACAGAATTAATACGACTCACTATAGGTTTTTTTTTTTTVN3′. For PCR, 1 μl of RT product was diluted three times and used as a template in each reaction. The reverse primer was from the adapter sequence 5′GCGAGCACAGAATTAATACGACTCAC3′, and the forward primers were specific to miR-15b and miR-16 mature sequences. U6 small noncoding RNA sequence was amplified as the internal control using the primers 5′GCTTGCTTCGGCAGCACATATAC (forward) and 5′TGCATGTCATCCTTGCTCAGGG3′ (reverse). The SYBR-Green-based real-time PCR was performed using the LightCycler 480 real-time PCR system (Roche Applied Science; Indianapolis, IN). The relative expression of miRNA was calculated based on the formula 2^(−Delta Delta Ct)^. Delta-Delta Ct values are Delta Ct_exp._ − Delta Ct_cont._.

### Western blot analysis

After rinsing with cold PBS, REC were collected in a lysis buffer containing protease and phosphatase inhibitors and scraped into tubes. Equal amounts of protein were separated on precast tris-glycine gels (Invitrogen, Carlsbad, CA), and then blotted onto a nitrocellulose membrane. After blocking in TBST (10 mM Tris-HCl buffer, pH 8.0, 150 mM NaCl, 0.1% Tween 20) and 5% (*w*/*v*) BSA, the membrane was treated with the appropriate primary antibodies followed by incubation with secondary antibodies labeled with horseradish peroxidase. Antigen-antibody complexes were detected by chemiluminescence reagent kit (Thermo Scientific, Pittsburgh, PA). Primary antibodies used were phosphorylated Akt (Serine 473), Akt, SOCS3, insulin receptor (all purchased from Cell Signaling, Danvers, MA), phosphorylated insulin receptor (tyrosine 1150/1151) (Enzo Life Sciences), insulin receptor phosphorylated on Tyr960 (Cell Applications, San Diego, CA), and beta actin (Santa Cruz, Santa Cruz, CA).

### ELISA analysis

A cleaved caspase 3 ELISA (Cell Signaling, Danvers, MA) was used to measure levels of the active apoptotic marker in whole retinal lysates. TNFα protein concentrations were measured using a TNFα ELISA (ThermoFisher, Pittsburgh, PA). For cleaved caspase 3 ELISA analyses, equal protein was loaded (60 μg) into all wells to allow for comparisons based on optical density (O.D.) For the TNFα ELISA, 100 μg protein was loaded into all wells, with analyses based on a standard curve. For phospho-IRS1 ELISA (Cell Signaling, Danvers, MA) analyses, 100 μg protein was loaded into all wells to allow for comparisons based on O.D.

### Statistics

Statistical analyses were done using Prism software (GraphPad, La Jolla, CA). Analyses were done using unpaired Student *t* test. Data are presented as mean ± S.E.M. For Western blots, a representative blot is presented.

## Results

### The levels of miR-15b and miR-16 expression are reduced in REC cultured in high-glucose conditions

**Figure 1:**
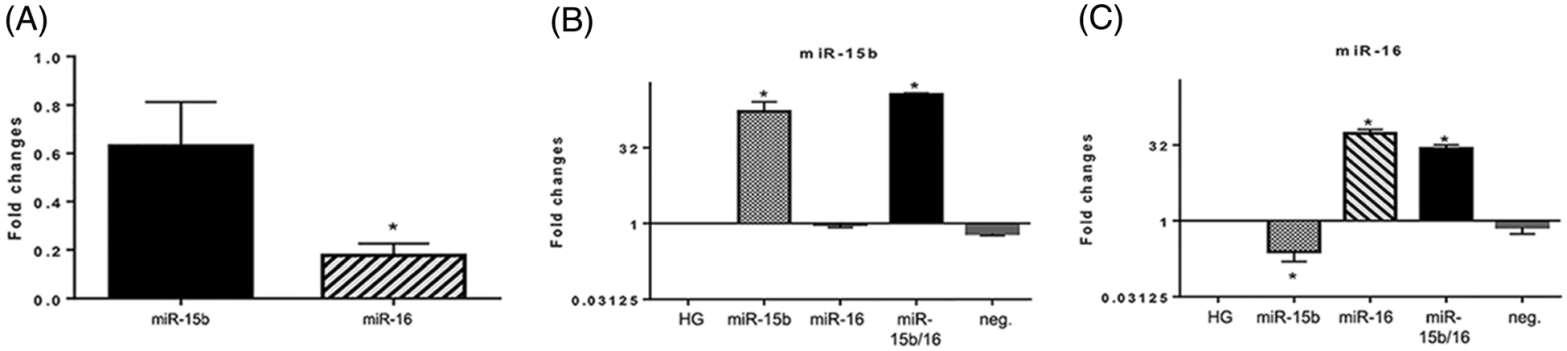
**Decrease of miR-15b and miR-16 expression in hyperglycemia and transfection-induced fold changes. (A)** Fold changes of microRNA (miR)-15b and miR-16 expression are shown. After 3 days of retinal endothelial cell (REC) culture in a high-glucose (25 mM) medium, the expression of both miR-15b and miR-16 was reduced (0.6- and 0.2-fold change, respectively) compared to that of the normal-glucose (NG; 5 mM) group. **(B, C)** Transfection-induced fold changes of miR-15b and miR-16 expression in REC. REC were transfected with mimics (30 nM of final concentration) of miR-15b and/or miR-16 to increase the level of expression in a hyperglycemic condition. Approximately 167- and 54-fold increases (miR-15b and miR-16, respectively) were detected following transfection with the mimic, compared to the high glucose (HG) control. The *y*-axis is a logarithmic scale. *P < 0.05 versus HG, *N* = 3; data are mean ± S.E.M.

We examined changes in miR-15b and miR-16 expression in REC after exposure to hyperglycemia. We cultured REC in a high-glucose medium (25 mM) and isolated the total RNA from the cells, followed by quantitative real-time PCR. We found that high glucose reduced the levels of miR-15b and miR-16, as compared to a normal glucose group (Figure 1A). Significantly decreased levels of miR-15b and miR-16, 0.6- and 0.2-fold change, respectively, were confirmed by quantitative real-time PCR.

Since hyperglycemia resulted in decreased expression of miR-15b and miR-16, we wanted to increase the miRNA expression through transfection with miRNA mimics. REC were transfected with mimics, miR-15b, miR-16, or miR15b + 16, at a final concentration of 30 nM for 48 h. Significant increases of the miRNA expression were confirmed by quantitative real-time PCR (Table 1 and Figure 1B, C). mRNA expression of miR-15b was increased by 167- and 364-fold, after transfecting with miR-15b and miR-15b + 16 mimics, respectively. The miR-16 expression was increased by 54- and 27-fold, following transfection with miR-16 and miR-15b + 16 mimics.

**Table 1:** **Fold changes of miR-15b and miR-16 expression after transfection with miR-mimics**

**Experimental groups**					
**miRNA**	**HG**	**miR-15b**	**miR-16**	**miR-15b/16**	**Negative control**
miR-15b	1.00	166.80	0.96	363.56	0.63
miR-16	1.00	0.24	0.24	27.33	0.75

*HG* high glucose, *miR* microRNA.

### miR-15b/16 reduced TNFα levels in hyperglycemia

**Figure 2:**
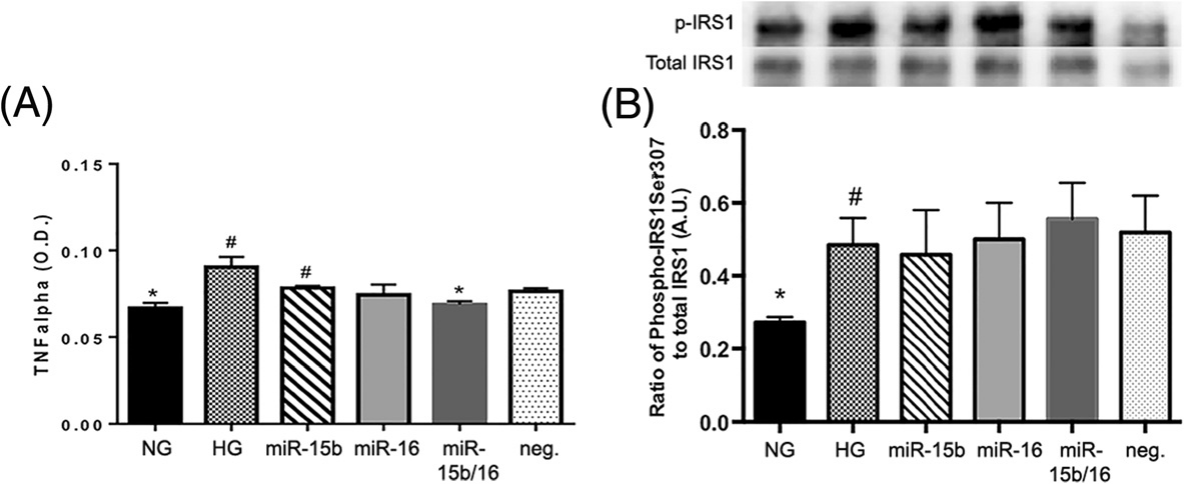
**Changes of TNFα and IRS1 (Ser307) levels in REC. (A)** Enzyme-linked immunosorbent assay (ELISA) data for tumor necrosis factor alpha (TNFα) on retinal endothelial cells (REC) in normal glucose (NG, 5 mM) or high glucose (HG, 25 mM) and transfected groups. microRNA (miR)-15b/16 decreased TNFα levels significantly compared to the control HG condition. **(B)** Western blot results for phosphorylated IRS1 on Ser307 and total IRS1. Increased levels of insulin receptor substrate 1 (IRS-1)^Ser307^ phosphorylation under hyperglycemic conditions were not changed in REC with miR overexpression. A representative blot is shown. ^#^ *P* < 0.05 versus NG, *P < 0.05 versus HG, *N* = 3; data are mean ± S.E.M.

Our goal was to determine whether miRNA-15b and miRNA-16 are involved in insulin signaling. Thus, we studied the effects of an altered miRNA expression on potential downstream signaling pathways known to be involved in diabetic retinopathy. We have previously shown that TNFα levels are increased in hyperglycemia [22]. We found that REC transfected with miR-15b/16 showed a significant decrease of TNFα levels, compared to a control HG condition (Figure 2A). We, therefore, demonstrated that the hyperglycemia-induced increase of TNFα levels were decreased in REC when miR-15b/16 are overexpressed. Additionally, we have previously reported that knockdown of TNFα led to a reduced phosphorylation of IRS-1 (Ser307), promoting normal insulin signal transduction [22]. In the present study, increased levels of IRS-1^Ser307^ phosphorylation under hyperglycemic conditions were not changed in REC with miR overexpression (Figure 2B). This suggests that miR-15b and miR-16 may work in REC activating other downstream signaling via TNFα. It is also possible that other potential microRNAs, which target IRS-1^Ser307^, counteract to maintain the phosphorylation of IRS-1^Ser307^ in hyperglycemia.

### miR-15b and miR-16 reduced SOCS3 levels in hyperglycemia

**Figure 3:**
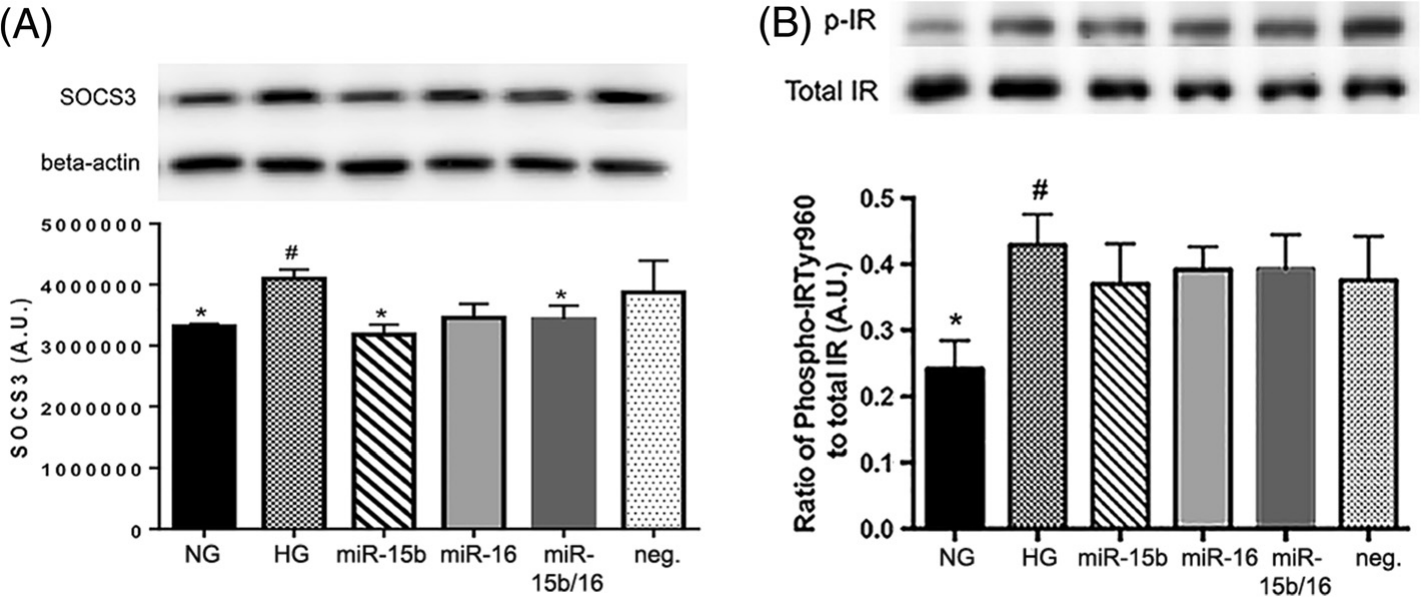
**The levels of SOCS3 and IR (Tyr960) phosphorylation in REC.** Western blot results for suppressor of cytokine signaling 3 (SOCS3) **(A)**, and insulin receptor (IR) (Tyr960) and total IR **(B)** on REC in normal glucose (NG, 5 mM) or high glucose (HG, 25 mM) and transfected groups. (A) The levels of SOCS3 were significantly reduced with overexpression of miR-15b and miR-15b/16 in hyperglycemia. (B) The levels of IR^Tyr960^ phosphorylation in HG were not affected by miR-15b and miR-16 overexpression. A representative blot is shown. ^#^ *P* < 0.05 versus NG, *P < 0.05 versus HG, *N* = 4; data are mean ± S.E.M.

Our previous work also demonstrated that TNFα increases SOCS3 signaling in REC [22]. Thus, we examined whether miR-15b and miR-16 alter SOCS3 levels in hyperglycemia. The Western blot data showed that high glucose significantly increased SOCS3 levels, as we expected. Overexpression of miR-15b and miR-15b/16 significantly reduced the levels of SOCS3 in hyperglycemia (Figure 3A). We, therefore, demonstrated that miR-15b and miR-15b/16 play a role in suppressing SOCS3 signaling in REC in hyperglycemia. Overexpression of miR-16 alone slightly reduced SOCS3 levels, although the change was not significantly different. Our previous study also showed that knockdown of SOCS3 decreased phosphorylation of IR^Tyr960^, which inhibited REC apoptosis through maintenance of normal insulin signaling [22]. In the present study, we found that the levels of IR^Tyr960^ in hyperglycemia were not affected by miR-15b and miR-16 overexpression (Figure 3B). This may indicate that miR-15b and miR-16 play a role in activating other downstream pathways via SOCS3.

### miR-15b and miR-16 increase the phosphorylation of IR^Tyr1150/1151^

**Figure 4:**
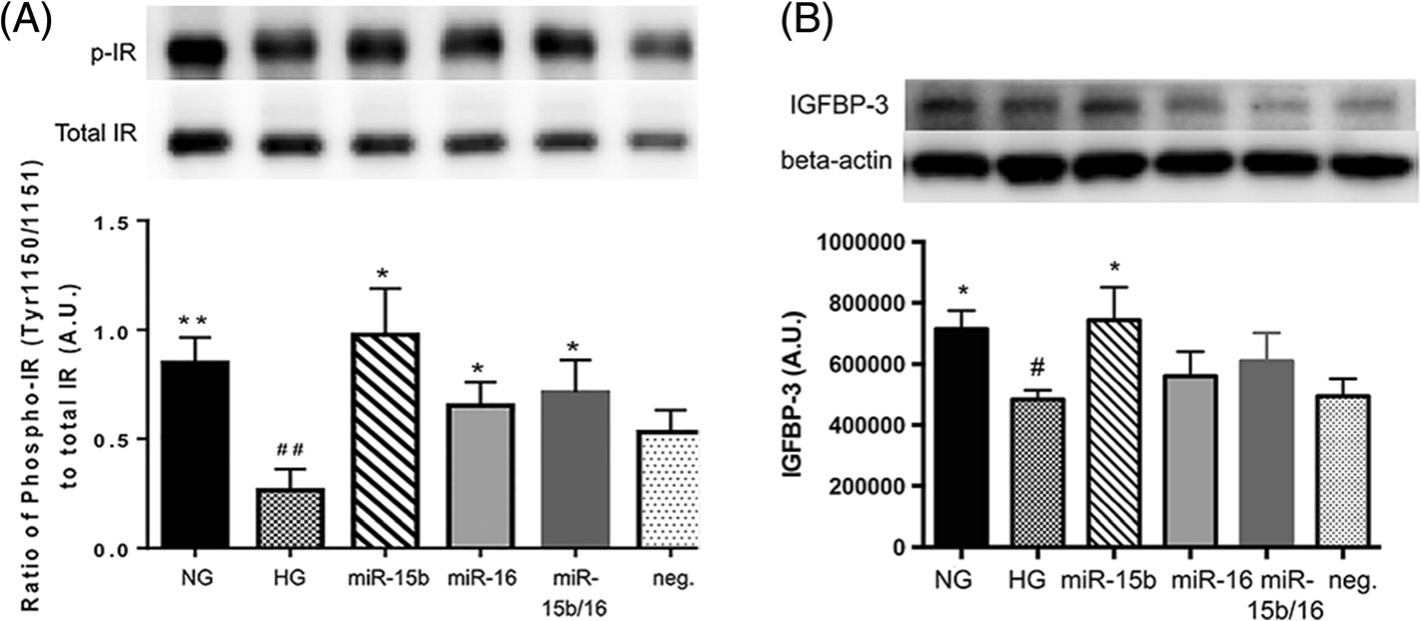
**Changes of insulin receptor (Tyr1150/1151) phosphorylation and IGFBP-3 levels.** Western blot results for phosphorylated insulin receptor (IR) (Tyr1150/1151) to total IR **(A)** and insulin-like growth factor binding protein-3 (IGFBP-3) **(B)** on retinal endothelial cells (REC) cultured in normal glucose (NG, 5 mM) or high glucose (HG, 25 mM) and transfected groups. (A) The levels of IR^Tyr1150/1151^ phosphorylation were increased by the overexpression of miR-15b and miR-16 in REC under hyperglycemic conditions. (B) miR-15b significantly increased the levels of IGFBP-3 in REC in hyperglycemia. A representative blot is shown. ^#^ *P* < 0.05 versus NG, *P < 0.05 versus HG, ^##^ *P* < 0.01 versus NG, **P < 0.01 versus HG, *N* = 6 ~ 7; data are mean ± S.E.M.

We previously reported that the increase of SOCS3 and TNFα, in hyperglycemia, is associated with increased levels of IRS-1^Ser307^ phosphorylation and decreased phosphorylation of IR^Tyr1150/1151^, leading to inhibition of normal insulin signaling [22,26]. In this study, we showed that miR-15b and miR-16 increased the levels of IR^Tyr1150/1151^ phosphorylation in REC cultured under hyperglycemic conditions (Figure 4A). The results indicate that the insulin receptor is one of the target pathways affected by miR-15b and miR-16 in REC, and elevated levels of the microRNAs protect REC in hyperglycemia.

In addition to increasing IR^Tyr1150/1151^, we recently demonstrated that insulin-like growth factor binding protein-3 (IGFBP-3) NB (a non-IGF-1 binding form of IGFBP-3) in diabetic rat retina reduced TNFα and SOCS3 levels and restored insulin receptor phosphorylation [27]. It has been demonstrated that IGFBP-3 has vascular protective effects [28,29], and IGFBP-3 has IGF1-independent effects, leading to the enhancement of cell survival [29-31]. In this study, we found that miR-15b in REC significantly increased the levels of IGFBP-3 in hyperglycemia (Figure 4B). That suggests that miR-15b may directly or indirectly target IGFBP-3, which contributed to the reduced signaling of TNFα and SOCS3, as well as increased IR^Tyr1150/1151^ phosphorylation to protect REC in hyperglycemia.

### miR-15b and miR-16 increase Akt phosphorylation and decrease apoptosis in hyperglycemia

**Figure 5:**
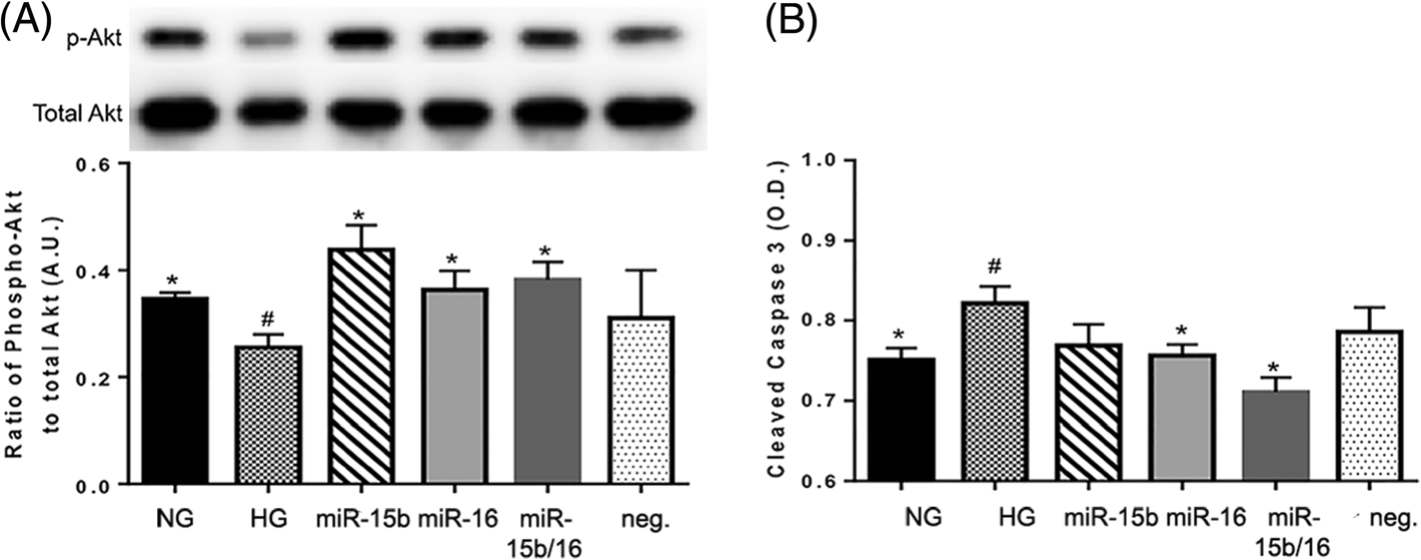
**Effects of miR-15b and miR-16 on Akt phosphorylation and cleaved caspase 3 in hyperglycemia.** REC were cultured in normal glucose (NG, 5 mM) or high glucose (HG, 25 mM). **(A)** Western blot results for phosphorylated Akt to total Akt on retinal endothelial cells (REC). Overexpression of microRNA (miR)-15b and miR-16 increased the levels of Akt phosphorylation, which was reduced in the control HG condition. A representative blot is shown. **(B)** Enzyme-linked immunosorbent assay (ELISA) results for cleaved caspase 3. miR-16 and miR-15b/16 significantly reduced the level of cleaved caspase 3 in hyperglycemia. ^#^ *P* < 0.05 versus NG, *P < 0.05 versus HG, *N* = 4; data are mean ± S.E.M.

It has been reported that reduced levels of TNFα can result in increased Akt phosphorylation, leading to a decrease of apoptosis [32,33]. Increased levels of cleaved caspase 3, resulting from the increased TNFα levels, cause cell apoptosis [34]. In hyperglycemic conditions, reduced levels of Akt phosphorylation in REC significantly increases pro-apoptotic proteins, including cleaved caspase 3 [22]. Therefore, we studied whether the increased expression of miR-15b and miR-16 could increase the phosphorylation of Akt in REC. We demonstrated that hyperglycemia reduced Akt phosphorylation, which was increased when miR-15b and miR-16 mimics were used to activate these specific miRNA (Figure 5A). No synergistic or additive effects were shown in the group with miR-combination, miR-15b/16, on Akt phosphorylation.

We also examined whether increased levels of miR-15b and miR-16 could decrease the cleavage caspase 3 of REC in a hyperglycemic condition. ELISA results showed that overexpression of miR-16 and miR-15b/16 significantly reduced the level of cleaved caspase 3 in hyperglycemia (Figure 5B). These results suggest that miR-15b and miR-16 can protect REC from apoptosis in hyperglycemia by activating the Akt survival pathway leading to reduced cleaved caspase 3.

## Discussion

Microvascular modifications are one of the significant alterations in diabetic retinopathy. Our previous studies and work of many others [35-37] have demonstrated that REC are one of the crucial cell types that are substantially affected in diabetic retinopathy, yet the cellular mechanisms underlying diabetic retinopathy are unclear. A new potential factor in diabetic retinopathy is regulation by miRNA. Our preliminary data and literature indicated potential roles of miR-15b and miR-16 in diabetic retinopathy [14,16,17]. Thus, in this work, we aimed to investigate changes in miR-15b and miR-16 in association with hyperglycemia-induced damage to cultured human REC. It has been reported that miR-15b/16 is expressed in multiple mammalian tissues, including the brain, heart, skeletal muscle, liver, lung, kidney, placenta, and spleen [38]. In the present study, we demonstrate the expression of miR-15b/16 in REC and show changes of the miRNA levels in response to hyperglycemia in human REC. Our results demonstrated that the expression levels of miR-15b and miR-16 were reduced in hyperglycemia. There is still only limited information on the effects of hyperglycemia with regard to miR-15/16. A study reported that high glucose did not cause changes of the level of miR-16 in mouse embryos *in vitro* [39]. However, others have shown that miR-15b was increased in the REC in STZ-induced diabetic rats, [14]. In addition to miR-15b and miR-16, other work has shown that miR-200b was downregulated in a high-glucose condition and played a role on glucose-induced VEGF upregulation in HUVEC [40]. Additionally, high glucose decreased the expression of miR-146a in HUVEC, which can function in regulating extracellular matrix protein production in diabetes [41].

Our previous work has shown that high glucose increases TNFα and SOCS3 protein levels in REC. In addition, increased levels of TNFα and SOCS3 lead to enhanced IRS-1 phosphorylation on serine 307, elevated IR (Tyr960), and decreased phosphorylation of IR (Tyr1150/1151), leading to insulin resistance and increased apoptosis [22,26]. In the present study, we demonstrated that an increased level of miR-15b and/or miR-16 in REC resulted in decreased signaling of TNFα and SOCS3, indicating the role of the microRNAs as regulators of these cytokine pathways in response to hyperglycemia. For the levels of IR (Tyr960) and IRS-1^Ser307^ phosphorylation in hyperglycemia, we did not find changes in these phosphorylated proteins after overexpression of mIR-15b/16. The results may indicate that miR-15b and miR-16 work in REC by activating other downstream molecules through TNFα and SOCS3 signaling, rather than targeting IRS-1 or IR^Tyr960^. Regulatory networks between miR and their targets are complex, since a single miRNA can bind to hundreds of target genes, and a target gene can be regulated by multiple miRNAs [42,43]. Each molecule at different levels of insulin signaling is targeted by a variety of miRNAs. Different miRNAs are involved in the expression of signaling proteins, such as insulin, insulin receptor, IRS-1 and IRS-2, and Akt [44]. It is possible that other potential groups of microRNAs, which target IR (Tyr960) and IRS-1^Ser307^, counteracted the effects of miR-15b and miR-16 on the inhibition of insulin signaling in REC cultured in hyperglycemia. It is reported that the number and abundance of miR targets decreases the ability of a single miR to suppress their targets [45]. Another possibility is that TNFα- and SOCS3-responsive miR (currently unknown) were activated in the signal transduction and this, in turn, interrupted the normal insulin signaling. Future work will be required to define the role of miR in TNFα and SOCS3 signaling.

In this study, we provide evidence that miR-15b and miR-16 may function in insulin signal transduction to protect REC during hyperglycemia, through increased IR^Tyr1150/1151^ phosphorylation. Moreover, we found that miR-15b and miR-16 increased the levels of IGFBP-3, whose expression was decreased in hyperglycemia. It has been reported that IGFBP-3 functions in an IGF1-independent manner, in addition to IGF1-dependent mechanisms, enhancing cell survival [29-31]. Our previous study has demonstrated that IGFBP-3 NB in diabetic rat retina reduced TNFα and SOCS3 levels and restored insulin receptor phosphorylation. In addition, IGFBP-3 directly reduced apoptotic markers, while increasing the anti-apoptotic marker Akt [27]. Different mechanisms underlying the independent effects of IGFBP-3 have been shown, including the activation of the insulin receptor/TNFα pathway [27], sphingolipid signaling [29], binding to IGFBP-3 receptor [46], and the involvement of protein kinase A [31] for the regulation of cellular apoptosis, as well as inactivation of the Erk1/2 pathway for anti-angiogenic and anti-tumor activities [47].

We demonstrated that REC overexpressing miR-15b and miR-16 were protected from hyperglycemia-induced apoptosis, showing increased levels of Akt phosphorylation with decreased cleaved caspase 3. Therefore, we provide a novel finding that miR-15b and miR-16 play a role in preventing insulin resistance in REC cultured in high glucose, via reduced activation of TNFα and SOCS3 pathways and increased IGFBP-3 levels.

Since miR-15b and miR-16 target many genes, including downstream molecules of insulin signaling, it is probable that signaling pathways, other than insulin signaling, are activated to maintain normal cell signaling in transfected REC in response to hyperglycemia. Little is known about the signaling pathways that are affected by miR-15b/16 in the retina. Investigating other potential pathways such as IGF-1-, integrin-, or growth factor-mediated signaling (predicted from *targetscan.org*) can be investigated to continue to increase understanding of the role of miR-15b/16 in diabetic retinopathy.

## Conclusions

Taken together, these results demonstrate that miR-15b and miR-16 play a role in the inhibition of insulin resistance via reduced TNFα and SOCS3 signaling and increased IGFBP-3 levels, resulting in REC protection from hyperglycemia-induced apoptosis. This outcome suggests that both miR-15b and miR-16 are potential therapeutic targets for rescuing diabetic retina. Therefore, these studies provide clues for the development of therapeutics for retinopathy and for understanding the roles of miR-15b/16 underlying the pathology of diabetic retinopathy.

## Abbreviations

ELISA: enzyme-linked immunosorbent assay
IGFBP-3: insulin-like growth factor binding protein-3
IR: insulin receptor
IRS-1: insulin receptor substrate 1
miR: microRNA
PCR: polymerase chain reaction
REC: retinal endothelial cells
SOCS3: suppressor of cytokine signaling 3
TNFα: tumor necrosis factor alpha
